# Lagging Strand Initiation Processes in DNA Replication of Eukaryotes—Strings of Highly Coordinated Reactions Governed by Multiprotein Complexes

**DOI:** 10.3390/genes14051012

**Published:** 2023-04-29

**Authors:** Heinz Peter Nasheuer, Nichodemus O. Onwubiko

**Affiliations:** Centre for Chromosome Biology, Arts & Science Building, Main Concourse, School of Biological and Chemical Sciences, Biochemistry, University of Galway, Distillery Road, H91 TK33 Galway, Ireland; n.onwubiko1@gmail.com

**Keywords:** genome stability, DNA replication, lagging strand DNA synthesis, Okazaki fragments, initiation, DNA polymerase α, DNA primase, CTC1-STN1-TEN1 complex, SV40 T antigen, CMG complex

## Abstract

In their influential reviews, Hanahan and Weinberg coined the term ‘Hallmarks of Cancer’ and described genome instability as a property of cells enabling cancer development. Accurate DNA replication of genomes is central to diminishing genome instability. Here, the understanding of the initiation of DNA synthesis in origins of DNA replication to start leading strand synthesis and the initiation of Okazaki fragment on the lagging strand are crucial to control genome instability. Recent findings have provided new insights into the mechanism of the remodelling of the prime initiation enzyme, DNA polymerase α-primase (Pol-prim), during primer synthesis, how the enzyme complex achieves lagging strand synthesis, and how it is linked to replication forks to achieve optimal initiation of Okazaki fragments. Moreover, the central roles of RNA primer synthesis by Pol-prim in multiple genome stability pathways such as replication fork restart and protection of DNA against degradation by exonucleases during double-strand break repair are discussed.

## 1. Introduction

Fidelity of DNA duplication is at the heart of preventing genome instability and associated diseases such as cancer and other genetic diseases [1,2,3]. DNA replication is essential for all living organisms. In all eukaryotes DNA replication is a highly conserved and tightly regulated process, which occurs once and only once per cell cycle in the synthesis phase or S phase of the eukaryotic cell cycle [4]. The DNA replication process can be divided into multiple steps: pre-initiation, initiation, elongation, and termination. Moreover, the linear nature of eukaryotic chromosomes creates a problem for their maintenance—the incomplete duplication of the telomeric ends on the lagging strand [1,2,3,5].

The understanding of DNA replication processes requires mechanistic knowledge about the coordination of the various multiprotein complexes involved in cellular DNA replication, from the origin activation, to double-stranded DNA (dsDNA) unwinding to form single-stranded DNA (ssDNA) templates, and the establishment of replication forks to the final DNA replication products. Generally, the DNA sequences, at which DNA replication is initiated first on a chromosome and at which replication fork(s) are established, are called origins of DNA replication [1,2,3,6,7]. After the separation of the two strands of the parental dsDNA, these newly established ssDNAs serve as templates for DNA polymerases, which synthesise the complementary strands. The polarities of these ssDNAs (5′-3′ and 3′-5′ when looking at the residues of the deoxyribose moiety) and the requirement of all DNA polymerase to synthesise nucleic acids in 5′ to 3′ direction (template direction 3′ to 5′) requires that at a given replication fork one strand, the leading strand, is synthesised in a continuous fashion whereas the other strand, called lagging strand, is synthesised discontinuously in the form of Okazaki fragments [1,2,3]. Here, it is important to note that eukaryotic DNA polymerases lack the ability to start DNA synthesis de novo and require a starter molecule, an RNA primer, for synthesising DNA. Whereas the continuous nature of leading DNA synthesis requires only one primer per replisome, the lagging strand synthesis requires an initiation event every one to two hundred nucleotides [1,2,3,8,9]. These primers are then further processed and maturated to yield the second continuous newly synthesised strand. Thus, understanding the mechanism and regulation of the initiation of DNA replication is central to the understanding of the DNA replication process in the eukaryotic cell cycle [1,2,3]. 

## 2. Initiation of DNA Replication at Origins

Each eukaryotic genome consists of multiple chromosomes, which in turn contain numerous origins of DNA replication, which regulate the unwinding of dsDNA and the start of semiconservative DNA replication, e.g., that replication of a given DNA is replicated precisely once per cell cycle [2,10]. They serve as functional organisers and each replicon or replication unit of a given chromosome contains one origin. Prior to the start of DNA replication in S phase, numerous coordinated early events occur. The pre-replicative complex is formed and involves origin recognition by the protein complex ORC (origin recognition complex, consisting of proteins ORC1 to ORC6), the loading of the MCM2 to MCM7 (minichromosome maintenance 2 to 7) proteins, the core of eukaryotic replicative helicases, with the help of CDC6 and CDT1/TAH11, their activation, and the formation of the pre-initiation complex [6]. These processes have been recently reviewed in detail [6] and are therefore not the focus of the current review. 

## 3. Replication Forks

The unwinding of dsDNA at eukaryotic DNA replication origin by the CDC45/MCM2-7/GINS (CMG) complex establishes two replication forks [1,2,3,6,7]. Each replication fork forms the junction between the chromosomal dsDNA and the two single-stranded template DNAs, leading strand and lagging strand. On the lagging strand, each Okazaki fragment is initiated by DNA polymerase α-DNA primase (Pol-prim) producing an RNA-DNA primer, which is then extended by DNA polymerase δ (Pol δ). In contrast, after the initiation by Pol-prim, DNA polymerase ε (Pol ε) synthesises DNA on the leading strand in a continuous manner [1,2,3,7,11].

a. CDC45/MCM2-7/GINS complex

The CMG complex is a very large protein complex and consists of the replication factor CDC45 plus the MCM2-7 and GINS complexes (the latter containing SLD5-PSF1-3) with MCM2-7 forming a hexameric ring around unwound ssDNA. CMG is the eukaryotic replicative helicase, which moves on the leading strand in 3′-5′ direction ahead of and in association with Pol ε. The enzyme complex unwinds dsDNA to provide the ssDNA templates for the replicative DNA polymerases [1,2,3,6,7]. Additionally, several proteins including AND-1/CTF4, Claspin/MRC3, Timeless/TOF1, and Tipin (Timeles interacting protein)/CMS3 associate with CMG and stabilise the replication fork, including linking them to replicative DNA polymerases [1,2,3,6,7,11,12].

b. The replicative DNA polymerases

b1. DNA polymerase α-DNA primase

**Figure 1:**
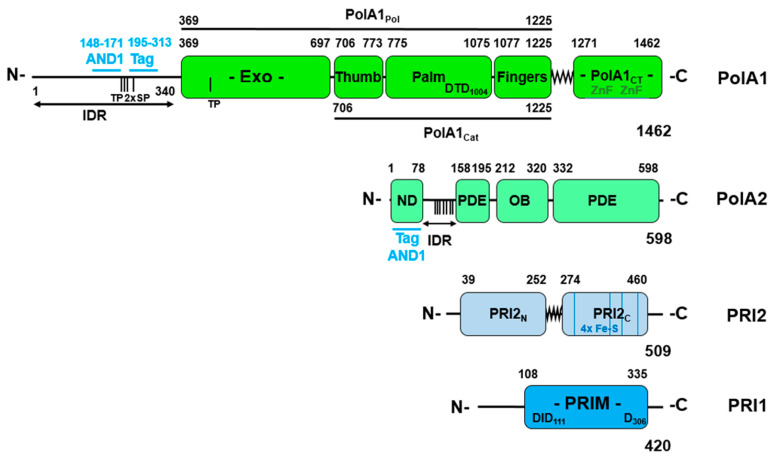
The four-subunit DNA polymerase α-DNA primase complex. The diagram shows the domain structure of the four subunits of DNA polymerase α-DNA primase (Pol-prim, [13,14,15,16]). The numbering follows the aa compositions of the human proteins [17,19,23]. The largest subunit PolA1 of Pol α (also named p180) contains an inactive exonuclease domain (Exo), the catalytic domains of DNA polymerase (Palm, Finger and Thumb, summarised as PolA1_Cat_), and a C-terminal domain PolA1_CT_. Exo and PolA1_Cat_ form the PolA1_Pol_ domain. PolA1_CT_ functions as an interaction site for the subunits PolA2 and PRI2, plus has DNA binding activity. PolA1_CT_ contains two Zn fingers (ZnF) and is connected to PolA1_Pol_ via a linker sequence (aa ~1226–1270), which forms different structures depending on the state of the enzyme complex. The N-terminus of PolA1 is an intrinsically disordered region (IDR, [18]), which interacts with other replication factors, e.g., AND-1/CTF4 and SV40 T antigen (Tag), and contains five in vivo phosphorylation sites, marked with short vertical lines, of which four are putative CDK sites (S/TP) [20,21,24]. PolA2 (B subunit, p68/p70) consists of an N-terminal protein–protein interaction domain (ND; AND-1/CTF4 and Tag binding), followed by an IDR having seven in vivo phosphorylation sites, highlighted with short vertical lines, of which six are putative CDK sites (**S**_126_**T**PE**T**PLTKR SVSTR**S**PHQL L**S**PSSF**S**PSA **T**P_158_, phosphorylation sites are in bold and underlined [20,21,24]). The subunit additionally contains inactive phosphodiesterase and OB-fold (oligonucleotide/oligosaccharide binding) domains [13,18]. The largest primase subunit PRI2 (p58, PRIL, PRIM2) is composed of an N-terminal and C-terminal domain, PRI2_N_ and PRI2_C_, respectively, which are connected via a flexible linker. PRI2_C_ also contains four Fe-S clusters presented as blue lines [13]. PRI1 (p48/9, PRIS, PRIM1) is the catalytic active primase subunit and contains three aspartates important to bind divalent cations (D109, D111, and D306), nucleotides, and ssDNA binding activities [13].

Pol-prim consists of four subunits: the large catalytic DNA polymerase subunit, PolA1 or p180, PolA2/p68/p70/B subunit, PRI2/Prim2/PriL/p58, and PRI1/Prim1/PriS/ p48/p49, the latter two forming the DNA primase complex (Figure 1, [13,14,15,16,17]). The N terminus of PolA1/p180 consists of an intrinsic disordered region (IDR, Figure 1, residue 1 to 340 [18]; these residue numbers use the sequence of human PolA1 [19]) and contain SV40 T antigen (Tag) and AND-1/CTF4-binding sites plus multiple CDK recognition sites [20,21,22]. This is followed by the polymerase domain (aa 369 to 1225, PolA1_Pol_), which has an inactive Exo domain, ssDNA binding and nucleotide binding activities, and is followed by a C-terminal domain (PolA1_CT_, aa 1271 to 1462). The latter functions as the platform for binding subunits PolA2 and PRI2; PRI2 is the large subunit of primase (Figure 1). The polymerase and the C-terminal domains are connected by a linker region (aa ~1226 to 1270, Figure 1 [13,14,15,16]). 

The second largest subunit PolA2 also called p68/p70 and B subunit binds to the C-terminus of PolA1. No catalytic activity has yet been assigned to PolA2, but it has regulatory function, e.g., it is phosphorylated by Cdk2 in a cell-cycle-dependent manner at its N-terminus, which also interacts with SV40 T antigen (Tag) and the human AND-1/CTF4 protein (Figure 1, [10,21,22,25,26,27]). PolA1 and PolA2 form the Pol α core complex [10]. The two smaller primase subunits PRI1 and PRI2, perform initiation functions [13,14,28,29,30]. PRI1 (small primase subunit PRIS, PRIM1, p48/9) carries the primase catalytic function, and PRI2 (large primase subunit PRIL, PRIM2, p58) is responsible for interaction with the large PolA1 subunit and regulating primer lengths. PRI2 is also required for stabilising the enzyme activity of the primase subunit of PRI1 in vitro [29]. PRI2 consists of an N-terminal, aa 39–252, a C-terminal domain, aa 274–460, and a hinge region, aa 253–273, connecting the two domains (Figure 1; [13,14]). 

b2. DNA polymerase δ 

DNA polymerase δ (Pol δ) is a heterotetrameric protein complex [1,2,11,31]. The largest subunit, p125, contains two enzymatic activities, a DNA polymerase and a highly active proofreading 3′ to 5′ exonuclease. Pol δ synthesises DNA with low processivity but PCNA (Proliferating Cell Nuclear Antigen), the processivity factor and replication clamp of Pol ε and δ, makes Pol δ a highly processive enzyme synthesising the main part of Okazaki fragments on the lagging strand. Pol δ also possesses efficient strand displacement activity and thus synthesises DNA beyond the RNA primer produced by DNA primase and may even remove most of the DNA primer polymerised by Pol α with the help of FEN1 and DNA2 protein [1,2,11,31].

b3. DNA polymerase ε

DNA polymerase ε (Pol ε) is also a four-subunit protein complex with the largest subunit, PolE/Pol2/p260, containing two enzymatic activities, a highly processive DNA polymerase, and a proofreading 3′ to 5′ exonuclease activity [1,2,11]. Although the Pol ε complex alone synthesises DNA with high processivity, PCNA enhances this processivity, allowing Pol ε the synthesis of a whole replicon size of DNA in one DNA synthesis cycle. At the replication fork, the leading strand replicase, Pol ε, physically and functionally interacts with CMG [11,32,33,34,35], e.g., stimulates the helicase activity of CMG [32,36]. Thus, PCNA, Pol ε, and CMG form a sandwich structure with Pol ε in the middle [11,32,35].

c. CMG-associated replication factors—members of the ‘replisome progression complex’

CMG has not only enzymatic function, e.g., helicase activity, but is also a central organiser of proteins at the replication fork [11,32,34,37,38,39]. It forms the logistic centre for association with other replication factors, e.g., the fork protection complex [32]. Here, AND-1/CTF4 forms homotrimers and recruits Pol-prim to CMG at the replication fork to increase Pol-prim concentration for enhancing the initiation of Okazaki fragment synthesis [22]. AND-1/CTF4 binds via its Sept B domain and HMG-box region to the N-termini of the two largest subunits of Pol-prim (Figure 1). Interestingly, the AND-1/CTF4 Sept B domain binds to the N-terminal IDR of PolA1 at position aa 148–171, which contains a CTF4 binding motif and is localised in close vicinity to the Tag binding site, aa 195–313 [18,22,38,40,41]. A second AND-1/CTF4 binding site in Pol-prim is localised in the N-terminus of PolA2, aa1-78, and overlaps with the Tag-binding site of PolA2 (Figure 1). These AND-1/CTF4 binding sites are not only close to or overlap with the Tag binding sites but may serve similar functions. The AND-1/CTF4-Pol-prim interactions could be important for the recruitment and loading of Pol-prim to the lagging strand for initiation [22,41,42]. Additionally, Mcm10, an essential replication factor, binds to the MCM2-7 complex and supports loading and stabilising Pol-prim at replication forks via its interaction with the N-terminus of PolA1 [1,2,3,11,43,44]. MCM10 also enhances the binding of Pol-prim to primers and thus may stimulate the hand-over of newly synthesised primers [45].

During unperturbed DNA replication, Claspin/MRC1 is necessary to maintain normal rates of replication fork progression [11,34,46]. To achieve this function, Claspin physically binds to DNA, especially to branched or fork-like DNA structures and connects the MCM2-7 complex with Pol ε, the leading DNA polymerase. Additionally, CMG interacts with a variety of proteins at the replication forks including Timeless/TOF1 and Tipin/CSM3 [11,34,38,46]. To stabilise replication forks during unperturbed and perturbed DNA replication conditions, Timeless/TOF1 and Tipin/CSM3 form a complex with CMG modulating the intra-S phase checkpoint and CHK1 phosphorylation. 

d. Additional factors supporting eukaryotic DNA replication

d1. Replication protein A 

Replication protein A (RPA) is heterotrimeric protein complex consisting of RPA70, RPA32, and RPA14, which tightly binds to ssDNA [2,47,48,49,50,51]. RPA’s binding stabilises ssDNA, prevents the formation of secondary structures within ssDNA sequences thus allowing smooth DNA synthesis by replicative DNA polymerase, and protects ssDNA against nuclease degradation. Beyond DNA replication, RPA is involved in all branches of eukaryotic DNA metabolism and DNA signalling after replication fork stalling towards ATR and at DNA lesions towards ATM and DNA-dependent protein kinase [2,47,48,49,50].

d2. Proliferating Cell Nuclear Antigen and Replication factor C

PCNA forms homotrimeric ring structures around DNA and is also known as sliding clamp [1,2]. As such, PCNA enhances the interaction of the replicative Pols δ and ε with the template primer allowing processive DNA synthesis. However, due to its ring shape, PCNA needs a helper function for loading on DNA. Replication factor C (RFC), the clamp loader, opens the PCNA ring in an ATP-dependent manner and loads it onto DNA. Additionally, RFC binds to DNA and the replicative DNA polymerases δ and ε [1,2].

### 3.1. Leading Strand Synthesis

At replication forks, on the leading strand, DNA primase synthesised the RNA primer that Pol α takes over and elongates to form an RNA-DNA primer. The latter, in turn, is taken over and extended further by Pol δ, before Pol ε starts the continuous DNA synthesis for the full replicon length in an unperturbed situation [1,2]. 

### 3.2. Lagging Strand Synthesis and the Initiation of Okazaki Fragment Synthesis

**Figure 2:**
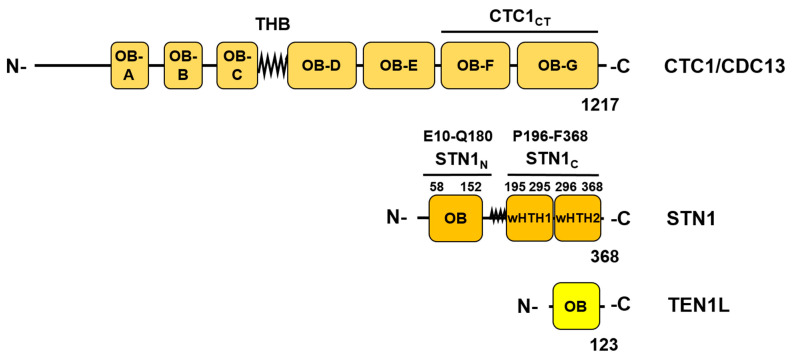
The domain structure of the heterotrimeric CST complex. The largest subunit of the CST complex, CTC1, also known as CDC13 and AAF132, consists of seven OB-fold domains and a hinge region [53,64]. The three helix bundle (THB) domain forms a hinge region between the three N-terminal OB-fold (OB-A to OB-C) and the four C-terminal OB-fold domains (OB-D to OB-G) with the two OB-fold domains OB-F and G (CTC1_CT_) being responsible for CST ssDNA binding and protein interactions within the pre-initiation complex (PIC). The second largest CST subunit, STN1 (AAF44), consists of an N-terminal domain STN1_N_ (E10 to Q180) mainly formed by an OB-fold domain (aa 58–152) and a C-terminal domain STN1_C_ (P196-F368) connected via a flexible linker. STN1_C_ contains two winged helix-turn-helix domains, wHTH1 and wHTH2 (aa 195–295 and aa 296–368, respectively). The smallest subunit TEN1/TEN1L comprises an OB-fold domain [64].

The synthesis of the lagging strand has major influences on the maintenance of eukaryotic genomes similar to leading strand DNA synthesis. Failures during the process of lagging strand synthesis can lead to increase in genome instability [52]. During the replication of a human genome, the replication machine must initiate 20 to 30 million Okazaki fragments. Despite its importance, relatively little is known about the initiation process of Okazaki fragments in eukaryotes. Recently, three eukaryotic model systems have shed more light on this central process: The lagging strand synthesis of telomere sequences uses Pol-prim and the CTC1-STN1-TEN1 (CST) complex (Figure 2; N.B in mouse CTC1 and STN1 are equivalent to the AAF132 and AAF44 subunits of alpha-Accessory factor (AAF) [53]). The SV40 system using Tag, RPA plus Pol-prim, and the cellular replication system with purified yeast and human proteins have also advanced our understanding of the lagging strand replication process [15,16,26,32,35,36,54,55,56,57,58,59,60,61,62,63].

a. Biochemical model systems for the initiation of Okazaki fragment synthesis

a1. Lagging strand synthesis initiation at telomeres 

The recent publication of four landmark articles has produced major advances in our understanding of the initiation of Okazaki fragment synthesis at telomere sequences and beyond on the mechanistic level and in general ([15,16,54,63] reviewed in [56]). Telomere sequences consist of repetitive DNA sequences at the ends of linear eukaryotic chromosomes and form specific protein–DNA complexes, the sheltering complex, to stabilise these ends during normal cell metabolism to avoid DNA damage signalling [5,65]. During chromosomal DNA replication, due to the removal of RNA primers on the lagging strand, telomere sequences become shorter during each round of chromosomal replication. Thus, special telomere replication processes exist in eukaryotic cells to extend these chromosomal end sequences [5,65]. The process consists of a telomerase-dependent G-strand synthesis forming an ssDNA extension with the repetitive sequence TTAGG followed by the formation of dsDNA by the synthesis of a complementary sequence, the second strand or C-strand. The latter requires the initiation of Okazaki fragments, which is carried out by Pol-prim in cooperation with the CST complex (Figure 2) as an essential auxiliary remodelling factor [15,16,53,54,63]. In human cells, CST tightly associates with Pol-prim, and both protein complexes copurify from human cell extracts [63]. During the replication of telomeric ssDNA, the CST complex binds to the single-stranded G-strand, modulates telomerase activity, and recruits Pol-prim to these telomeric sequences. Additionally, CST stimulates Pol-prim to initiate Okazaki fragment synthesis via its primase activity and synthesise the C-strand together with PCNA/Pol δ [63,66]. 

a2. CST remodels DNA polymerase α and DNA primase to initiate Okazaki fragment synthesis

**Figure 3:**
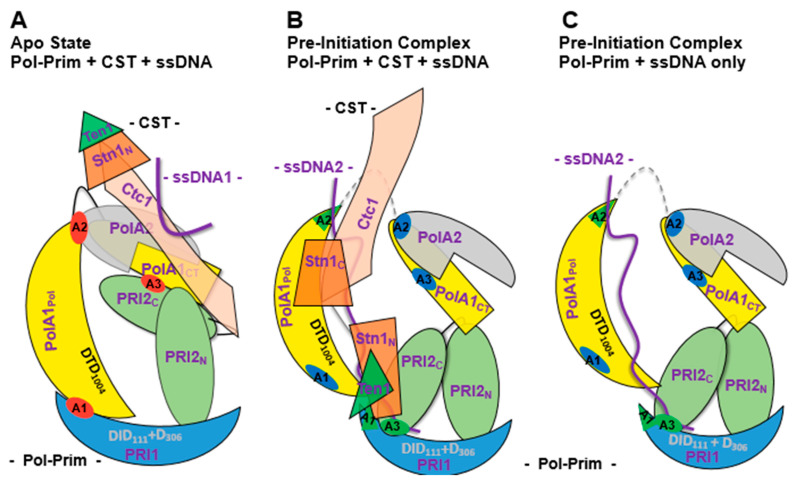
Rearrangements of the Pol-prim complex during activation of a closed APO form to an initiation-active enzyme. The diagram schematically summarises the remodelling of the Pol-prim complex architecture from a compact closed shape (inactive APO-state, (**A**) [15]) to an open form, the pre-initiation complex (PIC-state, (**B**,**C**), [16]) ready to synthesise RNA on ssDNA to initiate DNA synthesis. The (**A**,**B**) show Pol-prim associated with CST complex. To simplify the presentation and for clarity reasons the CST complex (**B**) was omitted in (**C**). In PIC, Pol-prim is shown in the presence of telomeric ssDNA (the latter is also included in the Pol-prim complex, (**C**)). It is important to note that especially the PolA1_cat_ and PRI2_C_ domains of Pol-prim show a high degree of movement in CryoEM studies [16]. The depicted structure is one of several states of Pol-prim in the active form, suggesting that multiple structures are associated with primase activity. Panel (**A**) only presents the STN1 N-terminus whereas the C terminus was not included in the structure structure 7U5C. In the diagram, PolA1 is shown in yellow (the PolA1 polymerase domain, PolA1_Pol_, is depicted as a half-moon with catalytic aspartates (DTD) highlighted, is linked to the C-terminal domain PolA1_CT_, top right in all panels, by a flexible hinge region). PolA1_CT_ acts as the binding site for the subunits PolA2, in grey, and the large primase subunit PRI2, shown in light green. The large primase subunit, PRI2 consists of an N- and a C-terminal domain (PRI2_N_ and PRI2c, respectively), whereas the catalytic RNA polymerase subunit PRI1 is presented as blue half-moon with the catalytic DID_111_ and D_306_ shown in grey (specifying that DID_111_ plus D_306_ are positioned away from the viewer). The red discs shown in panel A highlight the special interaction sites of PolA1_cat_ with PolA2 and PRI1 in the closed Apo complex, and also mark the APO state-specific PolA1_CT_-PRI2_C_ interaction, which are lost after the remodelling of Pol-prim into the open complex, the PIC. To show the distribution of these contact residues in the rearranged structure, they are again highlighted but in green or blue, as indicated in panels B and C. Interestingly, the binding site of PolA2 in PolA1 (Apo state) overlaps with a PolA1 DNA binding site in PIC (see panels B and C). In PIC, CTC1, STN1_N_ (both CST subunits), and PolA1_Pol_ bind to telomeric ssDNA (with CTC1 and PolA1_Pol_ surrounding the ssDNA and forming a tunnel whereas STN1 forms a passageway for the ssDNA template from PolA1_Pol_ to PRI1). Additionally, the C-terminus of the large primase subunit, PRI2_C_, binds to the ssDNA by flipping from its interaction with PolA1_CT_ in the Apo state to an association with telomeric ssDNA in PIC. Moreover, the two domains of PRI2 form together with PRI1 a small tunnel that allows directing the ssDNA template towards the catalytic triple aspartates D_109_, D_111_, and D_306_ of PRI1. (The diagram was assembled using the published structures 5EXR, 7U5C, and 8D0K, and information from [15,16,54,63]).

Biochemical and CryoEM studies of CST-Pol-prim complexes revealed an intricate mechanism of recruitment and activation of Pol-prim [15,16,63,67,68]. These findings suggest that in the first step, CST binds to the single-stranded telomeric G-strand via its CTC1 subunit and recruits Pol-prim via the C-terminal domain of PolA1 and both the N- and C-terminal domains of PRI2 to telomeric ssDNA (Figure 3A, [15]). At this stage, Pol-prim is in a closed, inactive form also called the APO state, similar to previously published structures of the free Pol-prim complex [15,16,69]. In this closed APO form, the polymerase domain of PolA1, PolA1_Pol_, binds to PRI1 and PolA2 subunit (Figure 3A, regions A1 and A2, highlighted in red [15]). In this complex, the PRI2 C-terminal domain, PRI2_C_, binds to the C-terminus of PolA1 (Figure 3A, binding site A3, highlighted in red), but not to PRI1, whereas the N-terminal PRI2_N_ domain independently binds to PolA1_CT_ and PRI1 (Figure 3A). These three protein–protein interactions A1 to A3 may contribute to inactivity of the Apo form of Pol-prim, as discussed later.

**Figure 4:**
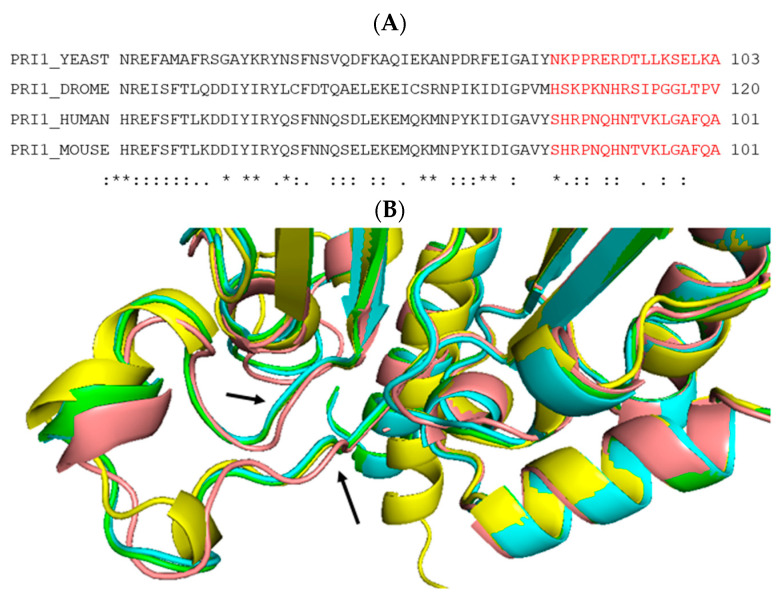
Conservation of the PRI1 loop binding to the Pol α subunit PolA1. In the APO form, the primase subunit PRI binds to the large subunit PolA1 with aa forming a loop: human and mouse sequences Y84-A101, yeast Y86-A103, and *Drosophila melanogastor* (DROME) H103–V120 (**A**). These residues are not well conserved from human to yeast at the sequence level (panel A shows a Clustal W alignment [70]; asterisks indicate identical aa in the PRI1 proteins of all four species, single and two points mark aa having low and high chemical similarity, respectively). In contrast, the three-dimensional structure of these PRI1 proteins is well conserved ((**B**). By using the PyMOL Molecular Graphics System, Version 2.0 Schrödinger, LLC, New York, USA), AlphaFold [71] predicted that structures were aligned. Arrows indicate the start and end residues of the PRI1 loop. Human PRI1 is in green, mouse in light blue, yeast in yellow, and *Drosophila melanogaster* in pink.

In the following, to establish primer synthesis, Pol-prim and CST form a pre-initiation complex (PIC). To this end, an extended rearrangement of the CST/Pol-prim complex and its interaction mode with ssDNA takes place (Figure 3B, [16]). The C-terminus of CTC1 forms an elongated complex on the G-strand (Figure 3B, ssDNA2) and the C-terminal domain of STN1, STN1_C_, (P196-F368) binds to CTC1 and PolA1_Pol_ (PolA1 polymerase domain aa 369–1225, Figure 1 and Figure 3). In contrast, Stn1_N_, the N-terminal domain of STN1 (E10-Q180, Figure 2), interacts with telomeric ssDNA, PRI1, and PolA1_CT_, the C-terminal domain of PolA1 (aa 1271 to 1462), with both STN1 domains being connected via the flexible hinge region P181-N195 (Figure 2 and Figure 3B). Additionally, in PIC, the smallest CST subunit, TEN1, contacts the primase subunits PRI1, PRI2_C_, and PolA2. Here, the PRI1 loop, Y84 to A101 in human PRI1 (Figure 4), which binds to a pocket formed in PolA1_Pol_ in the loading complex/Pol-prim Apo state, is located in the pocket formed by STN1_N_ and TEN1 (compare Figure 3A, red labelled A1 region, with Figure 3B, green label A1 in PRI1). Interestingly, the PRI1 loop Y84-A101 shows low degrees of sequence conservation from mammalian to yeast PRI1, whereas the loop structure is well conserved between the PRI1 proteins of these organisms (Figure 4A,B, respectively). It is also important to mention that in the ‘CST-Pol-prim loading complex’, Apo state of Pol-prim, large regions of CTC1 bind to Pol-prim via PolA1_CT_, PolA2, and PRI2, but neither STN1 nor TEN1 is involved in the complex formation. In contrast, in PIC, the smaller CST subunits closely engage with Pol-prim and ssDNA, but only ~300 amino acids of the C-terminal end of CTC1 bind Pol-prim and the ssDNA (Figure 2, compare structure 7U5C with 8D0K, summarised in Figure 3A,B). In PIC, the two C-terminal OB fold domains of CTC1, OB-F and G, and STN1_N_ bind to telomeric ssDNA in an oriented manner to recruit and remodel together with TEN1 the Pol-prim complex, arranging the latter in the right orientation on the template, and connecting the primase catalytic site in PRI1 with the DNA polymerase domain in PolA1_Pol_. ssDNA stabilises the link and the proteins plus ssDNA form together a pre-initiation complex, PIC (Figure 3B; [16]). Here, the high mobility of the PRI2_C_ domain seen in these two structures is discussed by He et al. (2022) [16]. This supports and highlights the existing knowledge that the PRI2_C_ domain is very mobile and involved in the handing-over of primer-ssDNA complexes to PolA2_Pol_. This is in line with previous reports that multiple interactions of PRI2_C_ with PRI1 support dinucleotide synthesis, whereas its association with PolA1 and PolA2 might be important for the hand-over of the newly synthesised primers to the catalytic centre of PolA1 [14,69].

This extensive remodelling of the Pol-prim complex during the transition from the CST-Pol-prim APO state to PIC, is highlighted by the fact that the interactions of PolA1_Pol_ with PRI1 and PolA2 are abolished and instead the region of PolA1_Pol_ interacting with PolA2 in the Apo state binds at least in part to telomeric ssDNA (Figure 3A,B, respectively, the red disc A2 in panel A is transferred into the green triangle A2 in panel B). In the APO state, PolA1 amino acids, H553-N555 and L645-Q649, interact with PolA2, whereas in PIC, the amino acids N552, Q554, K590, R616, Q649, R650, and N652-K655 bind to telomeric ssDNA in the CryoEM structure [15,16]. Here, Q554 and Q649 seemed to be crucial amino acids for both binding events in the PolA1-A2 and PolA1-ssDNA complex (compare structure 7U5C with 8D0K). The structure of Pol-prim in the Apo state [15] also suggests a possible resolution for activation of Apo state Pol-prim in the absence of remodellers such as CST, since the residues N652-K655 of the PolA1-ssDNA complex are relatively freely available in solution and binding of Pol-prim to ssDNA could initiate a transition of the Apo state into PIC by ssDNA itself. However, such a hypothesised binding of Pol-prim to ssDNA would most likely be less efficient than the formation of Pol-prim/CST/ssDNA PIC including the remodelling to initiate priming on telomeric ssDNA, natural ssDNA, and polydT templates in biochemical assays [63,67,68]. 

Additionally to its binding to PolA2 and CTC1, PolA1_CT_ interacts with PRI2_N_ and PRI2_C_ in the APO complex (Figure 3A). However, PolA1_CT_ stops interacting with PRI2_C_ when PIC is formed (Figure 3B,C). In contrast, PIC-PRI2_C_ establishes new physical interactions with PRI1 and PolA1_Pol_, which did not exist in the APO enzyme state (Figure 3). Moreover, in PIC, PRI1, PRI2_C_, and PRI2_N_ form a tunnel, which directs ssDNA towards the primase catalytic center in PRI1 (Figure 3B,C). The PRI1 loop, Y84-A101, which binds to PolA1 in the Apo state and associates with STN1_N_ and TEN1 in PIC, is part of this tunnel. In PIC, these two tunnels surrounding the ssDNA are connected by a passageway formed by STN1_N_ and ssDNA. These protein–ssDNA structures establish a tight grip on the telomeric G-strand in PIC, and direct the 3′-end of the ssDNA towards the catalyctic centre of PRI1 marked by the three aspartates D_109_, D_111_ and D_306_, which bind Mg^2+^ essential for nucleotide binding and the catalysis by primase. In the following, the coordinated binding of STN1_N_, PRI1, PRI2_N_, and PRI2_C_ allow the first di-nucleotide synthesis by primase. The importance of the rearrangement in PRI2 is underlined by the finding that mutations of the PRI2 linker region aa 256–270 result in a fivefold decrease in the di-nucleotide formation, whereas the ratio of di-nucleotide formation and longer primase products remains constant [13,14,69]. Interestingly, in PIC, the catalytic centre of the DNA polymerase subunit PolA1 marked by the catalytic aspartates DID_1002_ does not contact the ssDNA (Figure 3B). However, in the complex, the DTD_1002_ motif faces just towards the ssDNA in the STN1_N_–ssDNA passageway (Figure 3B) suggesting a mechanism by which PRI2_C_ and STN1_N_ hand over the primed G-strand to the PolA1 for primer extension and DNA synthesis. Thus, during the initiation of Okazaki fragment synthesis on telomere sequences, CST has recruitment and remodelling functions towards Pol-prim allowing the latter to initiate and synthesise the telomere C-strand. Interestingly, not all interactions seen in PIC are equally important. Adding STEN1 alone to biochemical assays is sufficient to stimulate the initiation activity of Pol-prim [67,72]. 

Importantly, physical and functional interactions of Pol-prim with CST and the remodelling of Pol-prim by CST are not only important for telomere C-strand DNA synthesis but also for genome-wide replication restart of stalled replication forks, e.g., in GC-rich sequences, which depends on CST [73,74]. Additionally, CST and Pol-prim cooperate to regulate the processing of double-strand breaks (DSBs), which are a major threat to genome stability in eukaryotes [75]. Here, 53BP1 modulates 5′-end resection at DSBs via fill-in synthesis performed by Pol-prim in complex with CST avoiding long 3′-overhangs and increasing fidelity of the DSB repair pathway, since long 3′-overhangs could be repaired by the single strand annealing pathway instead of homologous recombination, which may cause sequence deletions [76,77,78,79]. Additionally, the Pol-prim-dependent fill-in synthesis plays an important role in the efficiency of PARP inhibitors in BRCA1-deficient cells and breast cancer treatments [76].

The CryoEM structures discussed above together with biochemical data explain a mechanism for Okazaki fragment synthesis at telomere sequences (diagram in Figure 3) and raise the questions whether this mechanism can be extended towards the initiation process of Okazaki fragment synthesis at replication forks, and which proteins and protein complexes substitute for CST at replication forks. It is generally accepted that the heterotrimeric CST complex resembles structures and functions of heterotrimeric RPA, and CST even replaces RPA in replication fork restart after replication fork stalling to allow Pol-prim to initiate DNA synthesis [53,73,80]. Additionally, CST and RPA interact with Pol-prim, which supports the hypothesis that they recruit Pol-prim to ssDNA for the RNA-DNA primer synthesis. In contrast to CST, however, RPA does not stimulate Pol-prim on ssDNA templates and even diminishes the enzyme activities of the latter [72,81,82,83,84]. Thus, RPA might be a part of the Pol-prim remodelling activity on lagging strands at replication forks but it alone is not sufficient to remodel Pol-prim and transfer the latter from the Apo state to an enzyme equivalent to PIC-Pol-prim. Different activities and proteins independent of RPA or in collaboration with RPA have to substitute for this CST function during the initiation of Okazaki fragments. This view is supported by recent findings that the RPA subunit RPA32 stimulates Pol-prim on ssDNA templates whereas heterotrimeric RPA inhibits Pol-prim’s activity [72]. Interestingly, RPA32 exist as a free protein or in complex with other proteins in human cells and not only in association with RPA70 and RPA14 forming the heterotrimeric RPA complex. After depleting HeLa cell extracts of RPA70 together with associated RPA32, ~50% of RPA32 still remains in these RPA70-depleted extracts, suggesting that in mammalian cells RPA32 may have additional functions beyond those of the RPA complex (Rehmet and Nasheuer, unpublished results).

a3. SV40 T antigen, RPA, and DNA polymerase α-primase collaborate during the initiation of Okazaki fragment synthesis 

**Figure 5:**
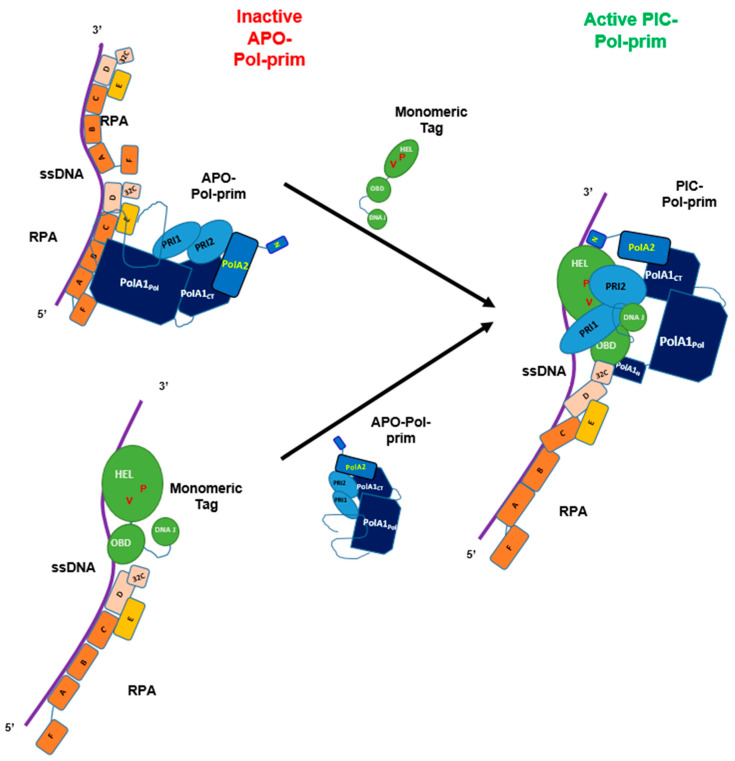
Functional interactions of SV40 T antigen, RPA, and Pol-prim during Okazaki fragment initiation on lagging strands of SV40 DNA replication. DNA primase does not start primer synthesis on RPA-bound ssDNA and requires the remodelling factor Tag to diminishing RPA-binding to ssDNA and enabling Pol-prim to synthesise RNA and DNA on ssDNA. In the SV40 lagging strand initiation model, the ssDNA is shown as a purple line; RPA consists of the large subunit RPA70 with the OB-fold domains A, B, C, and F shown in brown, the middle subunit RPA32 containing N-terminal phosphorylation sites, the central OB-fold D, and the protein interaction region RPA32C coloured in beige, plus RPA14 subunit, OB-fold domain E, in orange [48,91]); the Pol-Prim complex has the polymerase subunit PolA1 indicated in dark blue, consisting of an N-terminal Tag interaction region (PolA1_N_), the catalytic DNA polymerase domain (PolA1_Pol_), plus the C-terminal domain (PolA1_CT_) having multiple protein interaction sites, the regulatory subunit PolA2 in blue consists of the PolA2 N-terminal region (PolA2_N_) highlighted with an N that interacts with SV40 Tag, and the remaining PolA2 residues necessary for DNA replication, plus in light blue the two primase subunits, PRI1 containing the catalytic primase site, and PRI2 that interacts with PolA1 [12,13,26]); and the remodelling factor monomeric Tag in green, showing its three domains, the N-terminal DNA-J domain, the origin-binding domain (OBD), plus the helicase domain with V350 and P417 necessary for the stimulation shown as V and P, and highlighted in red-brown.

These studies of the lagging strand DNA synthesis initiation at telomeres give a novel insight into the mechanism of the initiation reaction of eukaryotic DNA replication and stimulate a fresh look at the initiation of Okazaki fragments at replication forks using established model systems. For a long time, the SV40 DNA replication system has served as an excellent model for human DNA replication including lagging DNA synthesis [85,86,87,88,89]. These studies have advanced our knowledge about mechanisms of the initiation of DNA replication including the Okazaki fragment synthesis at replication forks, e.g., by using SV40 T antigen (Tag), RPA, Pol-prim, and ssDNA in assay systems. Findings for the initiation of Okazaki fragment synthesis in the SV40 DNA replication system reveal similarities with those found for the C-strand synthesis at telomere sequences (Figure 5, [42,55,57,81,82,83,90]). 

In vivo and in vitro studies have revealed the multiple activities required to perform in SV40 DNA replication [87]. During the replication of dsDNA, SV40 Tag double-hexamers bind to and destabilise viral origin DNA sequences [85,89,92]. Then, after the origin unwinding, the two SV40 Tag helicase hexamers move in 3′ to 5′ direction on the leading strands of the replication bubble similar to CMG helicase [85,87,89,92], produce stretches of ssDNA, and load RPA onto ssDNA [87,90]. Next, SV40 Tag recruits Pol-prim to the replication fork to initiate DNA replication in the SV40 origin on the leading strand. Although RPA stimulates DNA polymerase activity of Pol-prim and acts as a fidelity clamp of Pol α DNA polymerase activity [93,94], during the initiation of DNA replication eukaryotic DNA primase cannot synthesise primers on RPA-bound ssDNA and requires additional helper functions to be active [57,81,82,83]. Here, Tag stimulates the primase and DNA polymerase activity of Pol-prim similar to human AAF/CST [53,57,63,67]. Thus, it is hypothesised that following the RPA loading onto ssDNA [87,90], the RPA-Tag complex directs Pol-prim, the priming enzyme complex, to ssDNA of the lagging strand (summarised in Figure 5; [48,49,57,81,82,83,90,95]). After producing an RNA-DNA primer, Pol-prim stops DNA synthesis and RFC transfers the primer with the help of PCNA to Pol δ to synthesise Okazaki fragments [87,89]. Interestingly, monomeric but not the hexameric form of SV40 Tag plays a role in stimulating the initiation reaction of Okazaki fragments (Figure 5; [57]). In the light of recent findings with telomere C-strand DNA synthesis (Figure 3), it is hypothesised that monomeric Tag forms a complex with RPA to direct Pol-prim to the template, and then Tag or the Tag-RPA complex acts as a Pol-prim remodeller opening the Pol-prim Apo complex and forming a pre-initiation complex consisting of Tag, RPA, and Pol-prim. This hypothesis suggests that this Tag-RPA complex would be the better functional equivalent for the Okazaki fragment synthesis at replication forks to the CST at telomere sequences than RPA alone (compare Figure 3A,B with Figure 5). However, at eukaryotic replication forks, two simultaneous or consecutive remodelling activities must occur during the initiation reaction. In the presence of RPA, the binding activity of RPA to ssDNA must be reduced, e.g., via an interaction of Tag with RPA32 C-terminus reducing the affinity of the neighbouring DNA binding domain OB-D to destabilise the RPA-ssDNA complex, plus Pol-prim must be transferred from its inactive APO form to PIC, similar as seen at telomeric ssDNA (compare Figure 3 and Figure 5) [63,81,82,83]. In the SV40 system, on the lagging strand, monomeric Tag takes over these functions as remodeller of Pol-prim and RPA [57,59]. 

In the model presented here, hexameric Tag has also important roles beyond its helicase activity at replication forks, e.g., functioning as recruitment and loading factor of Pol-prim similar as previously described for Tag in RPA loading during SV40 DNA replication [90]. Here, the interactions of Tag with the N-termini of PolA1 and PolA2 are important. Interestingly, Tag binds to the same regions in these two Pol-prim subunits as AND-1/CTF4, and CMG-AND-1/CTF4 complexes have similar supporting loading functions as hexameric Tag interactions in the SV40 system [22,32,38,41,87]. This interpretation is consistent structural biology findings showing that hexameric Tag binds via its ATPase domain to PolA2 N-terminus [26]. However, monomeric Tag is sufficient for the stimulation/activation activity on free and RPA-bound ssDNA suggesting that, at least under the conditions used, hexameric Tag is not required to stimulate Pol-prim [57,59]. The different requirements for the Pol-prim loading via the interaction of Tag with the N-termini of PolA1 and A2 and the remodelling during PIC formation and primer synthesis is supported by previous findings that species-specificity of initiation reactions of both, the origin-dependent initiation and Okazaki fragment synthesis of SV40 DNA replication, are regulated by the C-terminus of PolA1, residues K1149-S1462 [96]. The region contains binding sites for the small Pol-prim subunits including PolA2 and the linker region between the PolA1_Pol_ and PolA1_CT_ domain, but none of the known large Tag binding sites. These findings suggest that the interaction between PolA1 and the smaller subunits or the remodelling of the Pol-prim complex during the initiation reaction modulates the species specificity of SV40 DNA replication [26,40,96,97]. Thus, after loading Pol-prim onto RPA-monomeric Tag complexes on the lagging strand ssDNA, the Tag-helicase may stay associated with initiator Pol-prim since both N-termini flanking the Tag binding sites consists of IDRs [98], which are relatively flexible and adaptable to various conformations and having intrinsic flexibilities regarding distances of the interacting partners. Additionally, the hexameric Tag helicase may move on the leading strand, whereas Pol-prim initiates Okazaki fragment synthesis and elongates the primer on the lagging strand. This view is supported by data from multiple CryoEM experiments in which Pol-prim is not easily detectable at replication fork structures [99]. Similarly, He et al. describe multiple structures for Pol-prim in their CST-Pol-prim-ssDNA CryoEM data and have only analysed selected data sets in detail [16]. 

In the model, however, the hexameric Tag helicase at the replication fork would not activate Pol-prim on ssDNA and RPA-bound ssDNA but an additional Tag molecule, e.g., monomeric Tag, has a Pol-prim remodelling function [57,81,82,83]. Furthermore, previous findings also showed that Pol-prim changes from the APO structure to a PIC, and forming an initiation complex (IC, summarised in [13]) by binding to free ssDNA, but these ssDNA-driven rearrangements of Pol-prim are inefficient. Here, the binding of the PolA1_Pol_, PRI1, and PRI2 to ssDNA allow for opening the Pol-prim complex, e.g., by ssDNA competing with PolA2 binding to PolA1, and a basic enzyme activity is determined. However, the remodelling activity of Tag stimulates these key transitions of Pol-prim to an active enzyme by a 6 to 8 factor [57,59,82,83], similar to CST on telomeric ssDNA and other ssDNA templates [63,67,72]. Thus, in the model similar to CST, Tag may disrupt the PolA1_Pol_ interactions with PolA2 and PRI1 as well as diminishes the PRI2_C_’s physical binding to PolA1_CT_, allowing PRI2_C_ to form a small tunnel together with PRI2_N_ and PRI1 to stimulate the di-nucleotide synthesis by PRI1. PRI1 would then synthesise an oligoribonucleotide and, when it reaches the size of ~10 ribonucleotides, PRI2_C_ would hand over the primer to PolA1_Pol_, and the DNA polymerase activity of Pol α synthesises the DNA part of the RNA-DNA primer with a total length of ~30 nucleotides. Pol-prim then stops DNA synthesis and disintegrates from the template–primer system [81,83]. Next, RFC takes over the RNA-DNA primer and loads PCNA onto the primed ssDNA, which recruits Pol δ to processively synthesise a complete Okazaki fragment [87,89]. In parallel, Pol-prim, which is still attached to the replication fork via its link to the hexameric Tag helicase, will move towards the replication fork and start the synthesis of the next Okazaki fragment, as described above.

Since it is known that RPA and Pol-prim form direct physical interactions, alternatively, RPA recruits the Pol-prim in its Apo form to the lagging but the latter cannot start RNA primer synthesis in this complex with RPA and remains inactive (Figure 5, top part). Taking recent findings for the telomeric ssDNA into account, it is hypothesised that in next step, monomeric Tag remodels this Apo complex into PIC and then IC. The binding of RPA to ssDNA would also be remodelled by Tag’s interaction with RPA32C and interfering with the OB-D ssDNA binding activity [81]. Furthermore, upon Tag’s remodelling activity, interactions within the Pol-prim complex, e.g., PolA1_Pol_ with PolA2 and PRI1 are diminished and PRI2_C_ forms a tunnel structure with PRI1 and PRI2_N_ to allow the synthesis of the first di-nucleotide. Finally, PRI2_C_ would hand over the primed-lagging strand ssDNA to PolA1_Pol_, as described above.

a4. Elaborated activities of Okazaki fragment synthesis in yeast and human 

Understanding the replication of eukaryotic chromosomes has recently advanced with the establishment of biochemical replication systems using purified yeast and human proteins [36,58,62,99,100,101,102]. The yeast systems use origin-dependent replication, whereas in human systems, replication forks are established and fork-dependent DNA synthesis with the CMG complex and purified proteins is carried out. Both systems have been used to study leading strand DNA synthesis and they have also given new insights into the Okazaki fragment synthesis in eukaryotes [36,58,62,99,100,101,102]. Additionally, these purified proteins have been utilised for structural biological studies and single molecule studies [32,58,99,103]. Biochemical and structural biological experiments have shown that the eukaryotic replicative helicase, the CMG complex, plays a central role in the organisation of eukaryotic replication forks [32,36,102,104]. The biochemical assays showed the dependence and loading on the origin of yeast replication, and the leading strand synthesis with purified proteins at in vivo rates (reviewed in [11,104]) but the current review focuses on recent developments in understanding the initiation of Okazaki fragment synthesis. 

a5. Initiation of lagging strand synthesis at replication forks by Pol-prim

In eukaryotes, the findings from multiple replication systems suggest that the CMG complex is at the centre for recruiting and organising the protein activities at replication forks [62,100,101,103,104,105]. Here, Pol-prim is linked to CMG through the AND-1/CTF4 complex [41]. As recently shown, CDC45 of CMG is important for the loading of RPA on ssDNA. Thus, the CMG complex and associated proteins resemble multiple functions first described in SV40 T antigen-dependent DNA replication [57,87,106]. In recent single molecule studies, Lewis et al. shed light on another angle on the organisation of the eukaryotic replication forks [58]. They showed that a limited number of DNA polymerases interact with replication forks and that their exchange rate and numbers depend on the concentration of the polymerase. Low (5 nM) concentration of DNA polymerases showed a ratio of Pol ε-Pol δ-Pol α of 1-1-1 per replication fork, whereas at high concentration of ~20 nM the distribution was 1-1-2, respectively [58]. Taking into account that in vivo concentrations of these DNA polymerases in yeast culture is even above the higher concentration [107], one can assume that two Pol-prim molecules exist at replication forks in yeast cells. These findings raise the question of how these Pol-prim molecules are organised. Each subunit of AND-1/CTF4 homotrimer interacts with the IDRs of PolA1 and PolA2 N-termini (Figure 1), [18,98]. IDRs are relatively flexible and adaptable to multiple conformations allowing for flexibility in the distance of the interacting partners. Thus, via AND-1/CTF4 homotrimers, up to three Pol-prim complexes can attach to the CMG complex moving on the leading strand. This solves the conundrum, at least in part, that Pol-prim initiates Okazaki fragment synthesis and its DNA polymerase function elongates the RNA primer on lagging strands moving away from replication forks but remaining attached to CMG moving with the fork. These IDRs may function like ‘rubber bands’ allowing Pol-prim to associate with ssDNA substrate to synthesise the short RNA-DNA primer for Okazaki fragment synthesis moving away from the fork before releasing from the template and moving back to the fork for a new initiation event. It could be envisioned that one Pol-prim initiates the RNA primer synthesis including di-nucleotide formation, the rate-limiting step of the initiation reaction, close to the replication fork, whereas the second Pol-prim, slightly away from the fork, extends the dinucleotide to a short RNA and then forms RNA-DNA primers. This flexibility of the Pol-prim complex and its association with the replication fork is supported by the CryoEM data indicating that Pol-prim is difficult to localise at the replication forks [99], and that multiple Pol-prim complexes associate with CST at telomeric replication sites [16]. Here, two scenarios are envisioned: first, the CMG complex or associated proteins such as AND-1/CTF4 provide this Pol-prim remodelling activity and all happens at the replication fork simultaneously with loading of RPA and Pol-prim. Alternatively, the loading of RPA via the CMG complex is followed by the association of additional replication proteins, which would serve as remodelling factors for RPA decreasing its affinity to ssDNA and for Pol-prim to form an open complex capable of efficient primer synthesis. The latter opens the question of which replication protein(s) serve these functions during cellular DNA replication at replication forks. 

Regarding the initiation of Okazaki fragment synthesis at replication forks, a clear picture about the mechanism has not yet emerged and multiple scenarios are possible. In the first hypothesis, the CMG complex not only recruits and loads Pol-prim and RPA to the replication fork and onto the newly produced ssDNA, but also stimulates and remodels the Pol-prim complex to allow primer synthesis on the lagging strand, as seen in the primer synthesis on telomeric ssDNA. Having CMG as the major player also on the lagging with its movement on leading strand requires a complex mechanism for the coordination of the process, e.g., having multiple coordinated interactions between CMG, Pol-prim, and RPA allowing the primer synthesis by the primase subunits, and then support the handing over of the primed ssDNA to the catalytic centre of Pol α. Here, Mcm10 and AND-1/CTF4 are involved in loading and stabilising of Pol-prim at replication forks, but AND-1/CTF4 is not the remodelling cofactor stimulating Pol-prim activities since it does not enhance Pol-prim activity on unprimed M13 DNA [108]. Nevertheless, replication systems lacking additional factors are able to initiate leading and lagging strand synthesis in the presence of RPA. However, they use relatively high amounts of Pol-prim and the lagging strand synthesis products in these partially reconstituted systems are longer than in vivo products [8,9,36]. The addition of chromatin remodellers and histone chaperones, e.g., FACT and Nhp6, enhanced the lagging strand DNA synthesis [100,101] and yielded shorter Pol-prim primer products but they still do not reach the size of in vivo products as found in SV40-infected human cells and yeast cells [8,9]. These findings suggest that additional factors are most likely needed to supplement these replication systems. 

In an alternative view, the CST complex or variations thereof, e.g., the AAF subcomplex, not only carries out the remodelling of Pol-prim to initiate Okazaki fragment synthesis at telomere sequences, and restart replication synthesis after replication fork staling, but CST or subcomplexes thereof also stimulate Pol-prim during the initiation of Okazaki fragment synthesis on the lagging strand at replication forks. Such a hypothesis is supported by the copurification Pol-prim with CST from human cells, and the characterisation of Pol-prim stimulating factor AAF independently from the CST telomere function [53,63,109]. AAF is identical to a CTC1-STEN1 complex [80,110,111]. The characterisation of AAF showed that it stimulates both DNA primase and DNA polymerase activity of Pol-prim. It is important to note that mouse AAF and human CST efficiently stimulate Pol-prim on poly(dT)/dT72 templates [63,67,109]. Thus, these protein complexes are not only restricted to telomere G-strand or GC-rich sequences for Pol-prim stimulation [63]. Furthermore, the yeast and human STN1 protein have been shown to be sufficient to stimulate Pol-prim functions as well as the heterotrimeric CST complex [67,72]. Here, the N-termini of human and yeast STN1 are sufficient to stimulate Pol-prim on poly(dT) and telomere ssDNA sequences. Interestingly, in the Apo complex and the PIC, no interactions between STN1 and PolA2 were determined, but there are several additional unpublished Pol-prim complexes described, which have not been analysed in detail, and a crucial yet undescribed rate-limiting step for the stimulation activity may require STN1-PolA2 interactions [15,16,67,72]. Importantly, the interactions with PolA2 but not with ssDNA seem to correlate best with the stimulation of Pol-prim. Additionally, in human cells knocking down STN1/AAF44 also reduces the cellular DNA replication by ~50%. Moreover, STN1/AAF44 colocalises with PCNA in S phase cells [53]. However, genetic and cell-based assays have produced data that direct functions of Cdc13/CTC1/AAF132 and STN1/AAF44 towards telomere stability and replication-restart after replication fork stalling [5,73,112]. This apparent contradiction can be explained by the assumption that CST function is important for optimal replication but that other proteins, the CMG complex, or CMG-associated proteins such as MCM10, can in part substitute for CST function at replication forks, whereas CST is essential for telomere C-strand synthesis. This view is supported by findings that AND-1/CT-4 is not essential in yeast [38]. Importantly, omitting AND-1/CTF4 minimally increases the Okazaki fragment length [36]. Furthermore, increasing the Pol-prim concentration in the assay can rescue the omission of AND-1/CTF4 in the cell-free replication [36]. Such a redundancy in DNA replication functions has been previously reported for the Pol δ takeover of leading strand synthesis in yeast, carrying a catalytically inactive gene coding for Pol ε [113].

In a third hypothesis, other factors stimulate primase during Okazaki fragment synthesis at replication forks. Such proteins include free MCM2-MCM7; GINS; MCM4,6,7 complex; and RNase H, which have been described as factors that stimulate Pol-prim activity in biochemical assays and may allow optimal Okazaki fragment synthesis [114,115,116,117,118,119]. Additionally, RPA32 and STN1 have structural and functional similarities [53,80]. This is also true for the primase stimulation of STN1/AAF44 and it was shown that RPA32 stimulates Pol-prim primer synthesis with similar capacities as STN1, whereas the full RPA complex inhibits Pol-prim [72]. 

## 4. Outlook

The central roles of RNA primer synthesis in multiple genome stability pathways such as leading and lagging strand replication, replication fork restart, and protection of DNA against degradation by exonucleases have raised the interest into the mechanism of primer synthesis by Pol-prim. The stimulation of Pol-prim and Okazaki fragment synthesis in model systems using CST, STN1, RPA, and Tag are well characterised and understood using biochemical, molecular, and structural biological methods. Further studies to delineate the multiple steps to describe the process on a mechanistic level are still to come and will give new insights into this central process for the prevention of genome instability. Additionally, the roles of CST and Pol-prim in genome stability, such as replication fork restart and DSB pathways, are under intense analysis and exciting new insights in the cooperation of GST and Pol-prim are expected to be found in the near future. On the other hand, the initiation of Okazaki fragments at cellular replication forks, an important process which is involved in the replication of ~50% of the genome due to the contribution of lagging strand DNA synthesis, is partially understood and additional biochemical, genetic, molecular, and structural biological approaches will contribute to its understanding in the near future. Some new angles on this central process are discussed above and may contribute to solving the enigma of primase function on lagging strand synthesis at replication forks and beyond.

## Data Availability

No new data were created or analyzed in this study. Data sharing is not applicable to this article.
